# *Drosophila* VCP/p97 Mediates Dynein-Dependent Retrograde Mitochondrial Motility in Axons

**DOI:** 10.3389/fcell.2020.00256

**Published:** 2020-04-21

**Authors:** Ashley E. Gonzalez, Xinnan Wang

**Affiliations:** ^1^Department of Neurosurgery, School of Medicine, Stanford University, Stanford, CA, United States; ^2^Neurosciences Graduate Program, School of Medicine, Stanford University, Stanford, CA, United States

**Keywords:** VCP, p97, mitochondrial motility, Miro, dynein, live imaging, Drosophila, larvae

## Abstract

Valosin-containing protein (VCP), also called p97, is an evolutionarily conserved and ubiquitously expressed ATPase with diverse cellular functions. Dominant mutations in *VCP* are found in a late-onset multisystem degenerative proteinopathy. The neurological manifestations of the disorder include frontotemporal dementia (FTD) and amyotrophic lateral sclerosis (ALS). In these patients, long motor neuron axons could be particularly susceptible to defects in axonal transport. However, whether VCP has a physiological function in maintaining axonal transport and whether this role is impaired by disease-causing mutations remains elusive. Here, by employing live-imaging methods in *Drosophila* larval axons and performing genetic interaction experiments, we discover that VCP regulates the axonal transport of mitochondria. Downregulation of *VCP* enhances the retrograde transport of mitochondria and reduces the density of mitochondria in larval axons. This unidirectional motility phenotype is rescued by removing one copy of the retrograde motor *dynein heavy chain (DHC)*, or elevating *Miro* which facilitates anterograde mitochondrial movement by interacting with the anterograde motor kinesin heavy chain (KHC). Importantly, *Miro* upregulation also significantly improves ATP production of *VCP* mutant larvae. We investigate human *VCP* pathogenic mutations in our fly system. We find that expressing these mutations affects mitochondrial transport in the same way as knocking down *VCP*. Our results reveal a new role of VCP in mediating axonal mitochondrial transport, and provide evidence implicating impaired mitochondrial motility in the pathophysiology of VCP-relevant neurodegenerative diseases.

## Introduction

Neurons have exceptionally polarized axons and dendritic processes which enable rapid electrical transmission over a long distance. In humans, certain motor neuron axon terminals are as far as 1 m away from the cell body. Transporting essential organelles from the cell body to the axon terminal and sustaining the healthy organelles in each sub-compartment is a sophisticated challenge throughout the life of a post-mitotic neuron. Defects in axonal transport have been found in diverse neurodegenerative diseases including amyotrophic lateral sclerosis (ALS), Huntington’s disease, and Alzheimer’s disease (Baldwin et al., 2016; Sleigh et al., 2019). A recurring theme in those disorders is that longer axons are more vulnerable to disruptions of the transport of axonal cargoes. Therefore, a better understanding of the cellular mechanisms underlying axonal transport by disease-causing genes will help us uncover how disease mutations impair transport and cause neurodegeneration.

Allocating and sustaining a healthy pool of mitochondria in the extensive arborizations of neurons is especially important due to the multifaceted functions of mitochondria in providing ATP, buffering Ca^2+^, compartmenting metabolic reactions, and signaling cell death. The bidirectional movement of axonal mitochondria along microtubules is controlled by the anterograde motor kinesin that moves mitochondria from the soma to the axon terminals, and the retrograde motor dynein that transports mitochondria from the axons back to the cell body. Two mitochondrial-specific motor-adaptor proteins, Miro (RhoT1/2) and milton (TRAK1/2), play a key part in the regulation of anterograde transport of mitochondria. Miro is incorporated into the outer mitochondrial membrane (OMM) through Miro’s C-terminal transmembrane domain. Miro binds to milton which in turn binds to kinesin heavy chain (KHC), and in this way, Miro anchors mitochondria to motors and microtubules (Stowers et al., 2002; Guo et al., 2005; Wang and Schwarz, 2009b; Wang et al., 2011; Nguyen et al., 2014). When either *Miro* or *milton* is deleted in *Drosophila*, mitochondria remain in the soma and are unable to enter the axon (Stowers et al., 2002; Guo et al., 2005).

Valosin-containing protein (VCP), also called p97, is an evolutionarily conserved and ubiquitously expressed ATPase with diverse cellular functions (van den Boom and Meyer, 2018). The most-established function of VCP is to facilitate protein degradation through the ubiquitin-proteasome system where VCP binds to thousands of ubiquitinated proteins to assist their degradation by the proteasome. Additionally, VCP plays roles in signaling pathways, lysosome function, and autophagy. Dominant mutations in *VCP* cause a late-onset multisystem degenerative proteinopathy. The major clinical manifestations of the disorder include inclusion body myopathy (IBM), Paget’s disease of bone (PDB), frontotemporal dementia (FTD), and ALS. Despite the involvement of *VCP* mutations in multiple neurodegenerative conditions including motor neuron disease, a systemic evaluation of the role of VCP in axonal transport in an *in vivo* system is currently lacking.

The nervous system of *Drosophila* is an unsurpassed model to study axonal transport and human diseases. The vast collection of mutant lines and the ease of combining different mutants and transgenes in an intact organism enables robust genetic studies. The *Drosophila* genome shows a high degree of similarity to the human genome, and many fundamental regulatory processes of the nervous systems are conserved between humans and flies (Wang and Schwarz, 2009a). As a result, *Drosophila* has been successfully used to establish diverse human neurodegenerative disease models (Gunawardena et al., 2003; Clark et al., 2006; Park et al., 2006; Wang et al., 2007; Kim et al., 2013; Zhang et al., 2017). In *Drosophila* larvae, the cell bodies of central nervous system neurons are located in the ventral nerve cord. These cell bodies project motor neuron axons to the neuromuscular junctions in larval body wall muscles. We have established a live-imaging system that expresses fluorescent proteins in a subset of the neuronal axons in third instar larvae to study axonal transport of various cargoes (Wang and Schwarz, 2009a).

Fruit flies have one ortholog of *VCP* (*dVCP*), and knockout of *dVCP* is embryonic lethal (Hirabayashi et al., 2001). Mutations homologous to the human pathogenic mutations, *VCP-R155H* and *VCP-A232E*, have been introduced in *Drosophila*, as *dVCP-R152H* and *dVCP-A229E*, respectively (Ritson et al., 2010). While expressing wild-type *dVCP* in the neurons or muscles of flies results in no obvious phenotypes, expressing *dVCP-R152H* causes locomotor deficits, motor neuron death, and reduces survival (Kim et al., 2013). In this study, we live imaged mitochondria and dense core vesicles in *Drosophila* third instar larval axons and performed genetic interaction experiments to study the role of dVCP in axon transport. We demonstrated a physiological role for dVCP in regulating mitochondrial transport and the functional and pathological relevance of this role *in vivo*.

## Materials and Methods

### Fly Stocks

The following fly stocks were used: *Da-GAL4, w*^1118^ *(Wild-Type)*, *UAS-mCherry* (59021, Bloomington Drosophila Stock Center), *UAS-DHC*^64C[1–10]^ (8747, Bloomington Drosophila Stock Center), *UAS-GFP-RNAi* (41557, Bloomington Drosophila Stock Center), *CCAP-GAL4* (Wang et al., 2011), *UAS-T7-DMiro*^WT^ (Tsai et al., 2014), *UAS-White-RNAi* (a gift from Bingwei Lu), *UAS-VCP-RNAi1* (24354, Vienna Drosophila Stock Center), *UAS-VCP-RNAi2* (Zhang et al., 2017), *UAS-dVCP-WT*, *UAS-dVCP-R152H*, and *UAS-dVCP-A229E* (Ritson et al., 2010).

### qPCR

Total RNA was extracted from 20 third instar larvae by homogenization in TRIzol (Thermo Fisher) and mixing with chloroform vigorously. Samples were centrifuged at 12,000 *g* at 4°C for 15 min. The aqueous phase was then mixed with 100% isopropanol at 1:1 ratio to precipitate RNA. RNA pellets were washed with 70% ethanol, and then resuspended in nuclease-free water. 500 ng of total RNA was used to make cDNA using the iScript cDNA synthesis kit (BioRad) according to the manufacturer’s protocol. cDNA was mixed with TaqMan^®^ Gene Expression Assay Reagents (ThermoFisher) and analyzed by a Step One Plus Real-Time PCR System (Applied Biosystems). Each data point was normalized to the expression level of the housekeeping gene *RPL32*. The following primers were used (ThermoFisher):

DMiro: Dm02143924_g1

DHC: Dm01822116_m1

KHC: Dm01841230_m1

RPL32: Dm02151827_g1.

### Western Blotting

Adult flies (day 5) were lysed as previously described (Wang et al., 2011; Tsai et al., 2014). Lysates were analyzed by SDS-PAGE and immunoblotted with mouse anti-ATP5β (ab14730; AbCam) at 1:3000, mouse anti-β-actin at 1:3000 (ab8224; AbCam), guinea pig anti-DMiro (GP5) at 1:20,000 (Tsai et al., 2014), mouse anti-milton at 1:100 (Stowers et al., 2002), rabbit anti-VCP (SAB1100655; Sigma–Aldrich) at 1:5000, or rabbit anti-Marf (Ziviani et al., 2010) at 1:2000, and HRP-conjugated-goat anti-guinea pig, rabbit, or mouse IgG (Jackson ImmunoResearch Laboratories) at 1:5000.

### Live Image Acquisition and Quantification

Third instar wandering larvae were dissected in 1 × Ca^2+^ free saline (0.128 M NaCl, 2 mM KCl, 5 mM EGTA, 4 mM MgCl_2_, 5 mM HEPES, 0.0355 M Sucrose) at room temperature (22°C) in a chamber on a glass slide. Samples were imaged at room temperature with a 63 × /N.A.1.30 water Plan-Apochromat objective on a Leica SPE laser scanning confocal microscope (JH Technologies). For imaging motility, Mito-GFP or ANF-GFP was excited by a mercury lamp (HBO100), and images were captured every 2 s using a Leica DFC365 FX CCD camera for SPE II system with an I3 filter LP 515 nm (JH Technologies). Time-lapse movies were obtained continually with 2 s intervals for 100 s. For quantification, kymographs were generated from time-lapse movies by ImageJ. Each kymograph was then imported into a macro written in Labview (NI, TX, United States), and individual Mito-GFP or ANF-GFP puncta were traced using a mouse-driven cursor at the center of the GFP object. Using Matlab (The MathWorks, MA, United States), we determined the following parameters: (1) The instantaneous velocity of each mitochondrion, (2) the average velocity of those mitochondria that are in motion, (3) the percent of time each mitochondrion is in motion, (4) stop frequency, and (5) turn back frequency. The length of Mito-GFP or ANF-GFP puncta was measured using ImageJ from the first frame of the time-lapse series of images. All images were processed with NIH ImageJ using only linear adjustments of contrast and brightness.

### Immunostaining of Neuromuscular Junctions

Third instar wandering larvae were dissected in 1 × Ca^2+^ free saline (0.128 M NaCl, 2 mM KCl, 5 mM EGTA, 4 mM MgCl_2_, 5 mM HEPES, 0.0355 M Sucrose) at room temperature (22°C) on a sylgard coated dish. Larvae were incubated in 4% formaldehyde in PBS for 20 min at room temperature, and permeabilized overnight at 4°C (PBS + 0.3% Tween). Samples were immunostained with rabbit anti-GFP at 1:500 (A-11122, Invitrogen) overnight, then Alexa488-conjugated-donkey anti-rabbit IgG at 1:500 (711-545-152, Jackson ImmunoResearch Laboratories) and Alexa647-conjugated-goat anti-HRP at 1:100 (123-605-021, Jackson ImmunoResearch Laboratories) overnight. Samples were imaged with a 20 × /N.A.0.60 oil objective on a Leica SPE laser scanning confocal microscope (JH Technologies), with identical imaging parameters among different genotypes. Images were processed with ImageJ using only linear adjustments of contrast and color.

### Detection of ATP Levels

ATP levels were measured using a luciferase-based bioluminescence assay (ATP Determination Kit, 11699709001, Life Technologies). For each experiment, individual larvae were homogenized in 100 μl lysis buffer (provided by the kit). Lysates were boiled for 5 min, and cooled down on ice for 5 min. Then lysates were centrifuged at 14,000 r/min for 2 min. Supernatant was next diluted to 1:100 with reaction buffer (provided by the kit) and luciferase was added for 1 min at 25°C. The luminescence was immediately measured using a FlexStation 3 (Molecular Devices). Each reading was normalized to the protein concentration measured by a bicinchoninic acid (BCA) assay (23227, Thermo Scientific).

### Behavioral Assays

Crawling ability was defined as the distance larvae crawled in 60 s on a 60 mm grape agar plate poured over a 2 mm grid paper. Larvae were tested individually and given 10 s to acclimate to the environment. Videos were taken and scored blindly.

### Statistical Analysis

Throughout this paper, the distribution of data points is expressed as mean ± standard error of the mean (SEM), unless otherwise stated. Student *T*-test was used for statistical comparisons between two groups. One-way ANOVA *post hoc* Tukey test was performed for comparisons among multiple groups (adjustment applied) except otherwise stated. Statistical tests (one-sided) were performed using excel or SPSS.

## Results

### Downregulation of *dVCP* Alters Axonal Transport of Mitochondria

**FIGURE 1:**
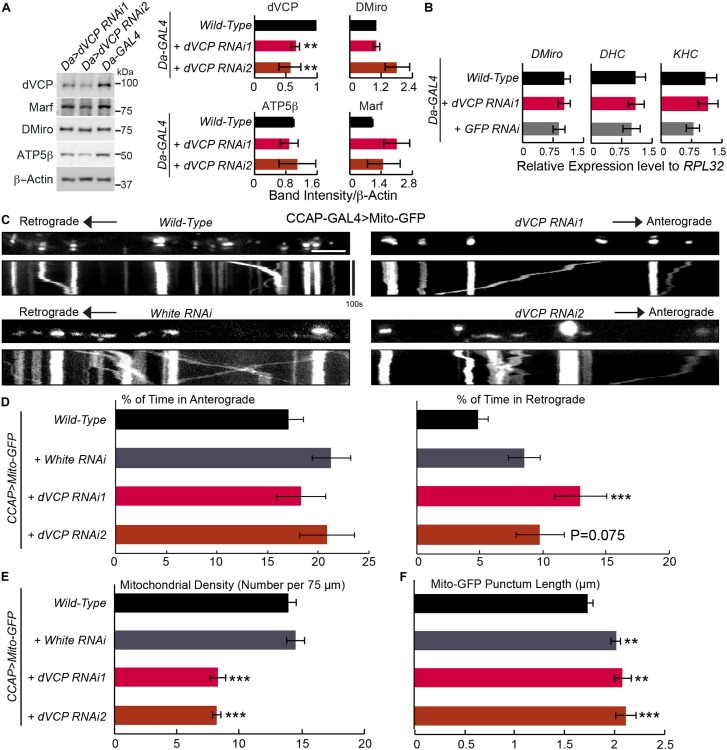
Knockdown of *dVCP* alters axonal transport of mitochondria. **(A)** Whole body lysates from adult flies five days after eclosion were immunoblotted as indicated. Protein band intensities are normalized to those of β-actin and graphed as relative change compared to “*Da-GAL4* > *Wild-Type*.” β-Actin levels are not significantly different among all genotypes. *n* = 3–6 independent experiments. **(B)** The mRNA expression levels were detected by qPCR on third instar larvae using primers amplifying genes as indicated, normalized to the expression levels of *RPL32* within the same experiment (*n* = 4 independent experiments). **(C–F)** Mitochondrial motility parameters are extracted from live imaging movies of mitochondria in larval CCAP-GAL4 expressing axons passing segment A3. **(C)** Mitochondrial movement labeled by Mito-GFP driven by CCAP-GAL4. The first frame of each live imaging series is shown above a kymograph generated from the movie. The *x*-axis of each kymograph is mitochondrial position, and the *y*-axis corresponds to time (moving from top to bottom). Vertical white lines represent stationary mitochondria and diagonal lines are moving mitochondria. **(D)** From kymographs as in **C**, the percent of time each mitochondrion is in motion in the anterograde and retrograde directions is determined and averaged (*n* = 164–334 mitochondria). **(E)** From kymographs as in **C**, the number of mitochondria present per 75 μm axon is quantified (*n* = 20–23 axons). **(F)** The length of each Mito-GFP punctum is quantified and averaged (*n* = 164–334 mitochondria). For **D–F**, 20–23 axons were imaged from 7 to 10 larvae per genotype. Comparisons with “*CCAP-GAL4* > *Mito-GFP, Wild-Type*”. Scale bar = 7.5 μm. **P < 0.01, ***P < 0.001, and error bars represent mean ± SEM here and for all figures.

In order to study the normal functions of dVCP, we ablated dVCP expression in flies. Because complete knockout of *dVCP* is embryonic lethal which does not permit imaging axonal organelles in larvae, we obtained two independent *UAS-dVCP* RNAi lines (Zhang et al., 2017). We employed the upstream activating sequence (UAS)-GAL4 system to turn on RNAi in a specific tissue (Brand and Perrimon, 1993). *dVCP* RNAi ubiquitously driven by the moderate driver Da-GAL4 allowed adult survivors. Using Western blotting, we confirmed partial reduction of dVCP protein expression by *dVCP* RNAi (Figure 1A). We found no significant changes of multiple mitochondrial markers including fly Miro (DMiro), milton, mitofusin (Marf), and ATP5β in *dVCP* RNAi mutant flies compared with wild-type controls (Figures 1A,B and Supplementary Figure S1A). These results are consistent with a recent report showing that in iNeurons with VCP inhibition, there is no drastic change in the abundances of OMM proteins including Miro and mitofusin (Ordureau et al., 2020).

To study the role of dVCP in mitochondrial transport, we live imaged mitochondrial movement in axons of third instar larvae. We labeled mitochondria with Mito-GFP in neuropeptidergic neurons driven by CCAP-GAL4, which allowed tracing individual mitochondrion moving in a single axon (Wang and Schwarz, 2009a; Tsai et al., 2017). We applied this live-imaging method to third instar larvae with *dVCP* RNAi. Notably, we found that the percentage of time axonal mitochondria spent moving in the retrograde direction was significantly increased in *dVCP* RNAi (line 1), and mitochondrial density in these axons was decreased compared to wild-type (Figures 1C–E). The velocities of moving mitochondria in either direction and the frequencies that mitochondria stopped or reversed directions were not significantly affected (Supplementary Figure S1B). Importantly, we observed similar phenotypes in a second *dVCP* RNAi line (line 2) (Figure 1). In this line, mitochondrial density was significantly decreased compared to wild-type, and the percentage of time mitochondria spent in moving in the retrograde direction was increased, although it did not reach statistical significance (*p* = 0.075). We confirmed that there was a lack of Mito-GFP in synaptic boutons at the neuromuscular junctions of *dVCP* RNAi (Supplementary Figure S1C), indicating that few mitochondria were delivered to the terminal. The supply shortage of presynaptic mitochondria may consequently disrupt synaptic innervations of muscles. To exclude the possibility of any artifact caused by our UAS-GAL4 system, we live imaged mitochondrial transport in axons with RNAi of the *white* gene, which determines the eye color of flies. By contrast, *white* RNAi did not affect mitochondrial density nor motility (Figure 1 and Supplementary Figure S1B), suggesting that the mitochondrial phenotypes we found in *dVCP* RNAi were caused by downregulation of *dVCP*. While there was no obvious mitochondrial accumulation or clumping (Figure 1C), the length of Mito-GFP puncta was slightly increased by *dVCP* RNAi (Figure 1F). However, because *white* RNAi also elongated Mito-GFP puncta (Figure 1F) and the increase in Mito-GFP punctum length by *dVCP* RNAi was very minor, this phenotype may not be biological meaningful. The unidirectional motility phenotype was not due to a change in the transcription of motor protein genes, as the mRNA expression of neither *KHC* nor *dynein heavy chain (DHC)* was influenced by *dVCP* RNAi as detected by quantitative real-time PCR (qPCR) (Figure 1B). In summary, *dVCP* deficiency causes mitochondria to spend more time in the retrograde direction and results in fewer mitochondria in the axons and synaptic boutons.

### *dVCP* Genetically Interacts With *Dynein* to Regulate Mitochondrial Transport

**FIGURE 2:**
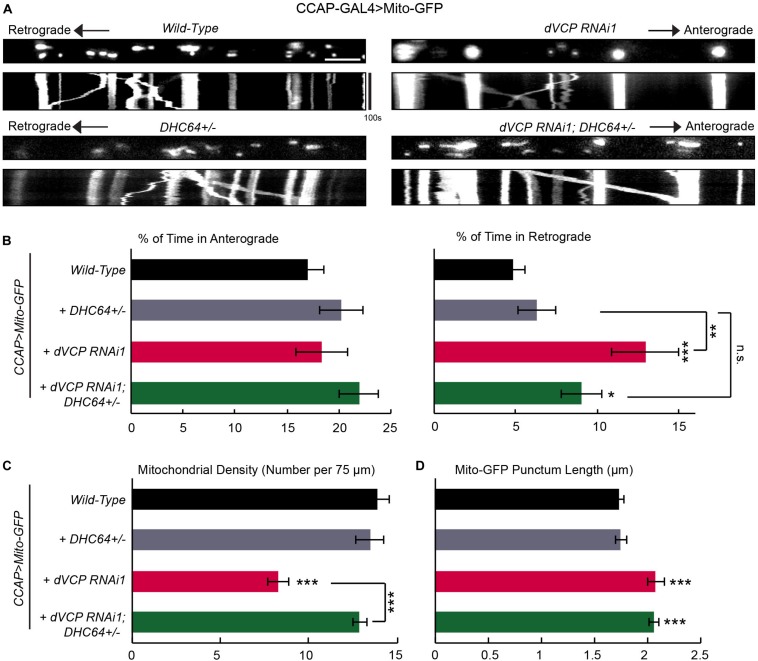
*dVCP* genetically interacts with *dynein* to regulate mitochondrial transport. **(A)** Mitochondrial movement labeled by Mito-GFP driven by CCAP-GAL4 in representative axons passing segment A3. The first frame of each live-imaging series is shown above a kymograph generated from the movie. **(B)** The percent of time each mitochondrion spends moving in the anterograde and retrograde directions is determined and averaged (*n* = 183–401 mitochondria). **(C)** The density of mitochondria (number per 75 μm) is shown (*n* = 13–31 axons). **(B,C)** Two-way ANOVA test reveals a significant difference between the impact of *DHC64C* +/− on wild-type and on *dVCP* RNAi for retrograde movement (*P* = 0.035) and density (*P* < 0.001). **(D)** The length of each Mito-GFP punctum is quantified and averaged (*n* = 183–401 mitochondria). For **B–D**, 13–31 axons were imaged from 6–12 larvae per genotype. Comparisons with “*CCAP-GAL4* > *Mito-GFP, Wild-Type*” (same data as in Figure 1) unless otherwise indicated. Scale bar = 7.5 μm. *P < 0.05, **P < 0.01, ***P < 0.001.

One potential cause of the unique motility phenotype in *dVCP* RNAi axons (Figure 1) was that the activity of the retrograde motor dynein itself was enhanced or the function of a mitochondrial dynein-adaptor was affected. If this was the case, mitochondria traveling in the retrograde direction would outnumber those in the anterograde direction, which may lead to fewer mitochondria left in the axons. To explore this possibility, we determined whether *dynein* and *dVCP* genetically interacted by removing one copy of *DHC* from the *dVCP* RNAi background (Gunawardena et al., 2003). Consistent with other reports (Gunawardena et al., 2003), keeping only one copy of *DHC* in a wild-type background (*DHC64* +/−) was sufficient to support mitochondrial movement and did not cause any phenotypes (Figure 2). However, removing one copy of *DHC* from the *dVCP* RNAi background prevented the phenotypes of increased retrograde mitochondrial transport and decreased mitochondrial density. This result confirmed that *DHC* and *dVCP* genetically interacted to regulate mitochondrial density and motility. Our findings suggest that dVCP may either inhibit the function of dynein or act through a dynein-adaptor to coordinate motor activities for transporting mitochondria in axons.

### Upregulation of Fly Miro Rescues Defects in Mitochondrial Transport Caused by *dVCP* RNAi

**FIGURE 3:**
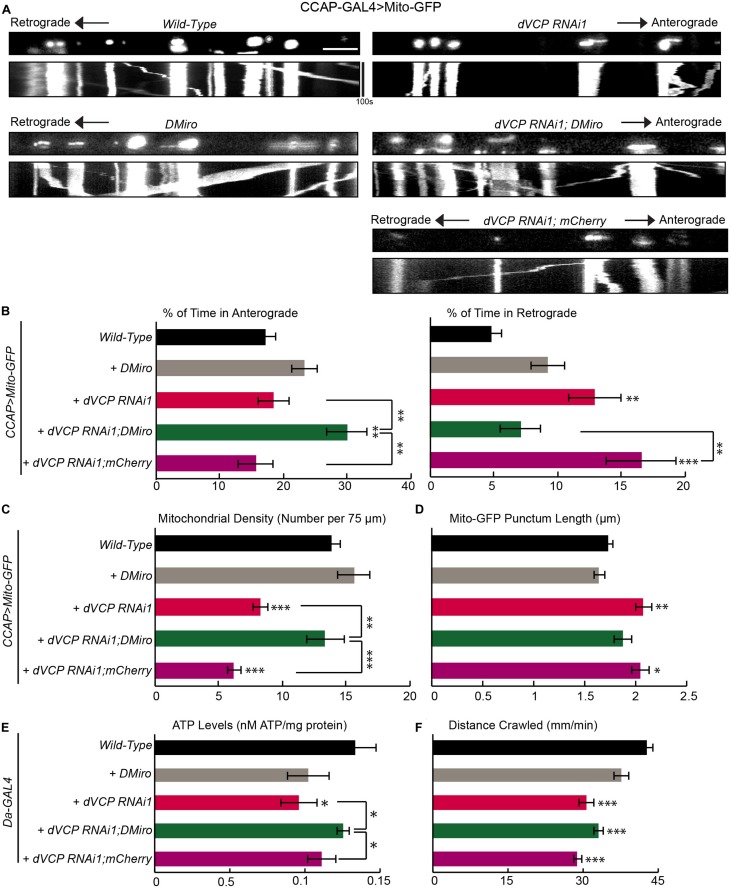
Overexpression of fly Miro rescues defects in mitochondrial transport caused by *dVCP* RNAi. **(A)** Mitochondrial movement labeled by Mito-GFP driven by CCAP-GAL4 in representative axons passing segment A3. The first frame of each live-imaging series is shown above a kymograph generated from the movie. **(B)** The percent of time each mitochondrion spends moving in the anterograde and retrograde directions is determined and averaged (*n* = 125–344 mitochondria). **(C)** The density of mitochondria (number per 75 μm) is shown (*n* = 12–28 axons). **(B,C)** Two-way ANOVA test reveals a significant difference between *DMiro*’s impact on wild-type and on *dVCP* RNAi for retrograde movement (*P* < 0.001) and near significant difference for density (*P* = 0.088). **(D)** The length of each Mito-GFP punctum is quantified and averaged (*n* = 125–344 mitochondria). For **B–D**, 12–28 axons were imaged from 6–12 larvae per genotype. Comparisons with “*CCAP-GAL4* > *Mito-GFP, Wild-Type*” (same data as in Figure 1) unless otherwise indicated. **(E)** Quantification of the total ATP level normalized to the protein concentration in third instar larvae (*n* = 5–6 larvae). **(F)** Larval locomotor ability was determined by the average distance in mm crawled in 60 s (*n* = 47–75 larvae). For **E,F**, comparisons with “*Da-GAL4* > *Wild-Type*” unless otherwise indicated. Scale bar = 7.5 μm. *P < 0.05, **P < 0.01, ***P < 0.001.

If the function of dynein or a dynein-adaptor was impacted in *dVCP* RNAi larvae and this caused more mitochondria to move out of the axons as our earlier results suggested (Figure 2), forcing mitochondria to re-enter the axons may also help rebalance the directional equilibrium of mitochondrial movement. To test this hypothesis, we overexpressed *DMiro* in *dVCP* RNAi larvae. DMiro has been shown to facilitate the anterograde mitochondrial transport by interacting with milton and KHC (Stowers et al., 2002; Glater et al., 2006). Indeed, upregulating *DMiro* in *dVCP* RNAi increased the percent of time mitochondria spent moving in the anterograde direction compared to that in *dVCP* RNAi alone. This approach also reduced the percent of time mitochondria spent moving in the retrograde direction, restored mitochondrial density, and shortened Mito-GFP punctum length to the wild-type level (Figures 3A–D). To exclude the possibility that the genetic backgrounds during the fly crossing rather than the *DMiro* transgene caused the rescue in *dVCP* RNAi, we expressed *mCherry* in *dVCP* RNAi, and we did not observe any rescue effect (Figures 3A–D). We next measured the ATP levels in third instar larvae with ubiquitous *dVCP* RNAi knockdown driven by Da-GAL4. *dVCP* RNAi reduced the ATP levels, and elevating *DMiro* significantly improved ATP production (Figure 3E). It is likely that redistributing mitochondria into axons and synaptic boutons by *DMiro* can rebalance gross energy homeostasis. Furthermore, *dVCP* RNAi third instar larvae were sluggish and slow in crawling (Figure 3F). This phenotype was not rescued by *DMiro* expression (Figure 3F). It is possible that behavioral improvements occur subsequently to mitochondrial functional improvements. Because the third instar larval stage is the final stage of larval development, our system may not allow us to see the long-term behavioral benefits of *DMiro* expression. Taken together, our results demonstrate that augmenting *DMiro* recalibrates the directionality of axonal mitochondrial movement and restores overall energy homeostasis in larvae lacking *dVCP*.

### Pathogenic Mutations of *dVCP* Alter Axonal Transport of Mitochondria Similarly as Downregulation of *dVCP*

**FIGURE 4:**
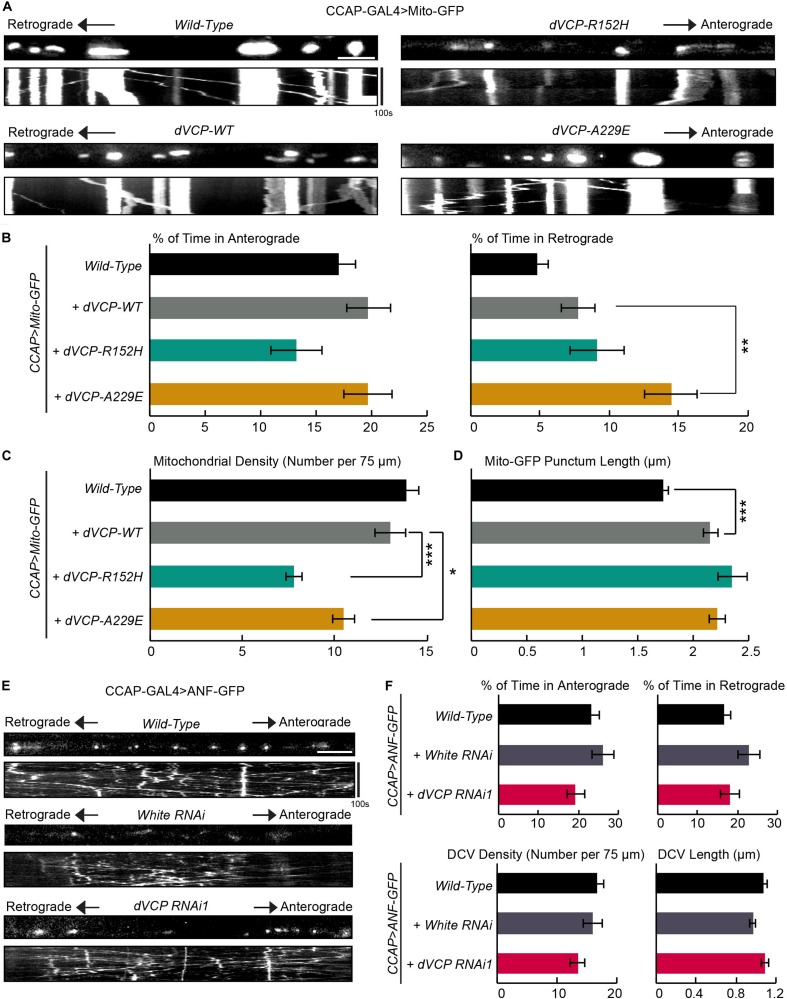
Pathogenic mutations of *dVCP* alter axonal transport of mitochondria similarly as downregulation of *dVCP*. **(A,E)** Organelle movement labeled by Mito-GFP or ANF-GFP driven by CCAP-GAL4 in representative axons passing segment A3. The first frame of each live-imaging series is shown above a kymograph generated from the movie. **(A)** Representative images and kymographs show mitochondrial movement. **(B)** From kymographs as in **A**, the percent of time each mitochondrion spends moving in the anterograde and retrograde directions is determined and averaged (*n* = 141–273 mitochondria). **(C)** The mitochondrial density (number per 75 μm) is shown (*n* = 18–28 axons). **(D)** The length of each Mito-GFP punctum is measured and averaged (*n* = 141–273 mitochondria). For **B–D**, 18–28 axons were imaged from 6–12 larvae per genotype. The data of “*CCAP-GAL4* > *Mito-GFP, Wild-Type*” are the same as in Figure 1. Comparisons with “*CCAP-GAL4* > *Mito-GFP, UAS-dVCP-WT*” as indicated. **(E)** Representative images and kymographs show movement of dense core vesicles from larvae of the indicated genotypes. **(F)** From kymographs as in **E**, the percent of time each dense core vesicle (DCV) spends moving in each direction, the density of DCV, and the length of DCV are calculated (*n* = 164–260 DCV). Comparisons with “*CCAP-GAL4* > *ANF-GFP, Wild-Type*.” Scale bars = 7.5 μm. *P < 0.05, **P < 0.01, ***P < 0.001.

We investigated whether human pathogenic mutations of *VCP* affected mitochondrial transport using our live-imaging system in fly larvae. We expressed wild-type *dVCP* (*dVCP-WT*), and mutant forms of *dVCP* (*dVCP-R152H* or *dVCP-A229E*) in CCAP-GAL4-driven larval axons together with Mito-GFP using the UAS-GAL4 system. We live imaged mitochondrial transport and analyzed movement parameters. We confirmed that both wild-type and mutant dVCP expressed at comparable levels by Western blotting (Supplementary Figure S1D). Interestingly, while *dVCP-WT* had little effect on mitochondrial transport, both *dVCP-R152H* and *dVCP-A229E* decreased mitochondrial density in axons (Figures 4A,C), similar to *dVCP* RNAi (Figure 1). *dVCP-A229E* also significantly increased the percent of time mitochondria traveled in the retrograde direction (Figures 4A,B), like *dVCP* RNAi (Figure 1). Both wild-type and pathogenic *dVCP* subtly increased Mito-GFP punctum length (Figure 4D), although there was no obvious mitochondrial aggregation (Figure 4A). Therefore, mutations homologous to human pathogenic mutations, especially A229E, act on mitochondrial motility and density in a dominant negative manner.

### Downregulation of *dVCP* Does Not Affect Axonal Transport of Dense Core Vesicles

Because dynein is a motor shared by multiple cargoes, we determined whether other cargoes were similarly affected by *dVCP* RNAi. Using our imaging system in larvae, we live recorded dense core vesicles labeled by ANF-GFP in axons. In contrast to the phenotypes of mitochondria, we did not detect any defect in the movement, density, or length of ANF-GFP puncta in *dVCP* RNAi (Figures 4E,F). These results suggest that dVCP specifically regulates mitochondrial transport, likely through a mitochondrial dynein-adaptor, rather than dynein itself.

## Discussion

In this study, we have revealed a crucial role of dVCP in regulating axonal mitochondrial motility *in vivo*. We have provided genetic evidence linking *dVCP* to the *dynein* motor, and have shown that rebalancing the directions of mitochondrial movement is beneficial for mitochondrial function. Importantly, we have demonstrated that mutations homologous to human pathogenic *VCP* impact mitochondrial transport in a dominant negative fashion in flies, adding a new layer of understanding to VCP-linked diseases.

Although VCP is a well-known master regulator of diverse cellular functions and processes, VCP’s importance in axonal transport has not been studied. This is a missed opportunity, because *VCP* mutations are found in ALS, where defective mitochondrial transport has emerged as a central theme in the pathology of the disease (Granatiero and Manfredi, 2019). Our findings are in line with previous reports implicating the significant involvement of trafficking deficits in the etiology of motor neuron disease (Baldwin et al., 2016; Granatiero and Manfredi, 2019). Interestingly, overexpressing *Miro* has been shown to rescue phenotypes of mitochondrial motility in a SOD1 cultured neuron model of ALS (Moller et al., 2017). In our VCP fly model, the defects of mitochondrial transport are not identical to those in the SOD1 model, yet upregulation of *Miro* is beneficial in both models (Figure 3). Similarly in a SOD1 mouse model of ALS, reducing Parkin has been found to delay the loss of Miro, slow motor neuron loss, and prolong survival (Palomo et al., 2018). These findings indicate that Miro may be a converging therapeutic target in genetically distinct ALS/FTD patients. Importantly, we have previously shown that small molecules removing Miro from damaged mitochondria rescue neuron loss and improve locomotor abilities in Parkinson’s disease models (Hsieh et al., 2019). Therefore, pharmacologically manipulating Miro seems to have great potential for treating multiple neurodegenerative diseases.

VCP has been shown to regulate mitofusin levels and mitophagy (Kim et al., 2013; Zhang et al., 2017). In our experimental settings, multiple mitochondrial proteins including Miro, mitofusin, milton, and ATP5β are unaltered in *dVCP* RNAi (Figure 1A and Supplementary Figure S1A), suggesting that mitophagy is intact. Consistent with our results, a very recent paper has shown when VCP is inhibited in iNeurons, the protein abundances of OMM proteins including mitofusin and Miro are not dramatically affected (Ordureau et al., 2020), indicating that VCP does not play a significant role in controlling OMM protein turn over. The different results are likely caused by differences in the experimental systems. Alternatively, VCP may have distinct roles in different tissues at different developmental stages. In high energetic tissues such as adult flight muscles (Zhang et al., 2017), VCP’s role in mitophagy may be more important than in other tissues.

Long-term neuronal homeostasis and survival rely heavily on a sustained supply of healthy mitochondria in each micro-domain during the lifetime of a neuron (Wang and Schwarz, 2009a; Course and Wang, 2016). The ability of dVCP to orchestrate motor functions could be essential to maintaining synaptic structure and transmission, particularly at the terminals of long axons. We have revealed that an unidentified mitochondrial-specific dynein-adaptor works in concert with dVCP to modulate mitochondrial transport. Elevating the mitochondrial kinesin-adaptor Miro may enhance the function of kinesin and antagonize the activity of the dynein complex when dVCP is missing (Figure 3). These findings support a model where individual motors and adaptors on the same cargo coordinate and influence each other’s actions. Our study adds a new branch to the already complex biological functions of VCP and emphasizes the importance of VCP in neuronal physiology.

## Data Availability Statement

The datasets generated for this study are available on request to the corresponding author.

## Author Contributions

AG designed and performed the experiments, made the figures, and wrote the manuscript. XW conceived and supervised the project, designed the experiments, and wrote the manuscript.

## Conflict of Interest

The authors declare that the research was conducted in the absence of any commercial or financial relationships that could be construed as a potential conflict of interest.
