# Supplementary material for: Drosophila VCP/p97 Mediates Dynein-Dependent Retrograde Mitochondrial Motility in Axons
Source: Front Cell Dev Biol. 2020 Apr 21;8:256. doi: 10.3389/fcell.2020.00256 (PMC7186335; doi:10.3389/fcell.2020.00256)

Drosophila VCP/p97 Mediates Dynein-Dependent Retrograde Mitochondrial Motility in Axons.

Ashley E. Gonzalez[1,2], and Xinnan Wang[1]*

Affiliations:

1. Department of Neurosurgery, 2. Neurosciences Graduate Program, Stanford University School of Medicine, Palo Alto, CA 94304, USA.

* Correspondence: xinnanw@stanford.edu

Supplementary Information

Supplementary Figure 1. *dVCP* RNAi Phenotypes and Transgenic dVCP Expressions. (A, D) Whole body lysates from adult flies five days after eclosion were immunoblotted as indicated. Protein band intensities are normalized to those of β-actin and graphed as relative change compared to "*Da-GAL4>Wild-Type*" for (A) and "*Da-GAL4>dVCP-WT*" for (D). β-Actin levels are not significantly different among all genotypes. (A) n=3 independent experiments. (D) n=5 independent experiments. (B) The average velocity of non-stationary mitochondria in each direction is shown (n=32–128 mitochondria). The frequency that each mitochondrial instantaneous velocity changes to zero (stop), and that the direction of the instantaneous velocity reverses (reverse) is quantified (n=164–334 mitochondria). 20–23 axons were imaged from 7-10 larvae per genotype. (C) Representative confocal stack images show the membranes of neuromuscular junction boutons visualized by anti-HRP and mitochondrial puncta visualized by mito-GFP at

muscle 12 hemisegment A3 in third instar larvae. Similar results were seen in 3 larvae per genotype. Scale bar=30 µm.

Supplementary Figure 1

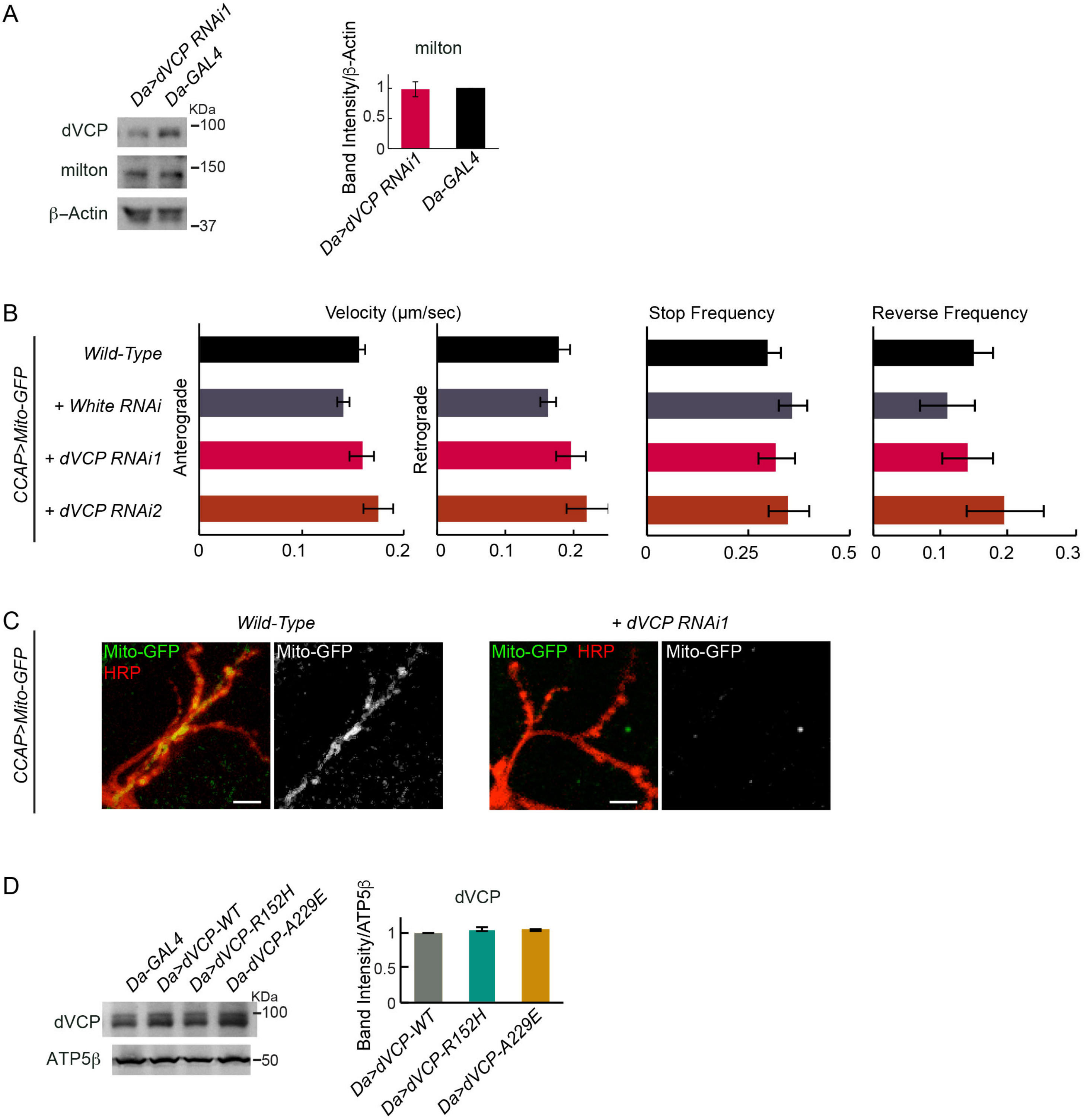

Supplement: Supplementary file 1 [file Data_Sheet_1.pdf]
